# How muscle contraction strengthens tendons

**DOI:** 10.7554/eLife.44149

**Published:** 2019-01-24

**Authors:** Nicole O Glenn, Clarissa A Henry

**Affiliations:** 1 Department of HematologySt. Jude Children's Research HospitalMemphisUnited States; 2 School of Biology and EcologyUniversity of MaineOronoUnited States; 3 Graduate School for Biomedical Sciences and EngineeringUniversity of MaineOronoUnited States

**Keywords:** tendon, tenocyte, mechanotransduction, mechanical force, myotendinous junction, Danio rerio, Zebrafish

## Abstract

The force generated by muscles leads to signaling that helps to shape nearby tendon precursor cells.

**Related research article** Subramanian A, Kanzaki LF, Galloway JL, Schilling TF. 2018. Mechanical force regulates tendon extracellular matrix organization and tenocyte morphogenesis through TGFbeta signaling. *eLife* **7**:e38069. doi: 10.7554/eLife.38069

The Chinese classic text Tao Te Ching states that “A journey of a thousand miles begins with a single step”. However, this famous proverb does not consider the impact of these steps on the unsung heroes of the musculoskeletal system – the tendons that connect muscle to bones and enable the skeleton to move. Tendon injuries (such as tendonitis, tendinopathy, and tendon rupture) are painful and difficult to heal. Typically caused by overuse, and especially frequent in weightlifters, such injuries occur because the tendon does not adapt as readily to changes in tension as muscle does.

Tendons are fibrous extracellular matrices (ECMs) composed of collagen, proteoglycans and other glycoproteins. In mature tendons, these ECM proteins are secreted by tendon cells. One reason that tendon injuries heal slowly is that tendons have relatively few of these cells (Lui, 2015). Currently, there are no efficient therapies for tendon injuries because it is not well understood how tendons respond to muscle contractions. Now, in eLife, Thomas Schilling of the University of California, Irvine and colleagues – Arul Subramanian (UC Irvine) as first author, Lauren Kanzaki (UC Irvine), and Jenna Galloway (Massachusetts General Hospital) – demonstrate that developing zebrafish embryos are excellent models for studying how tendons adapt to forces (Subramanian et al., 2018). This is because zebrafish embryos have perfected the art of maintaining functional tendons during dramatic growth of the musculoskeletal system.

Muscles and tendons work as an integrated unit to transmit force to the skeletal system and stabilize joints, and there is clear evidence that they depend on each other to develop correctly. In mice, muscles and tendons develop during the same time period and the formation of tendon progenitor cells depends on the presence of early muscle in mouse embryos (Brent et al., 2005). In zebrafish, disrupting muscle development has detrimental effects on the structure of tendons, and disrupting the tendon results in abnormally long muscle fibers (Hall et al., 2007; Goody et al., 2010).

Little is known about how mechanical force affects tendon development, even though force plays an important role in tendon repair. Subramanian et al. therefore established a way to image how tendon precursor cells (also known as tenocytes) develop. This technique uses transgenic zebrafish in which the regulatory elements for a tenocyte-specific gene called *scxa* control when the fluorescent protein mCherry is expressed (Schweitzer et al., 2001; Chen and Galloway, 2014).

Subramanian et al. observed that muscle contraction helps to shape the tenocytes (Figure 1). The tenocytes elongate and extend protrusions perpendicular to the muscle cells. Unexpectedly, these protrusions are microtubule-based rather than the actin-based protrusions more frequently observed in cells.

**Figure 1.**
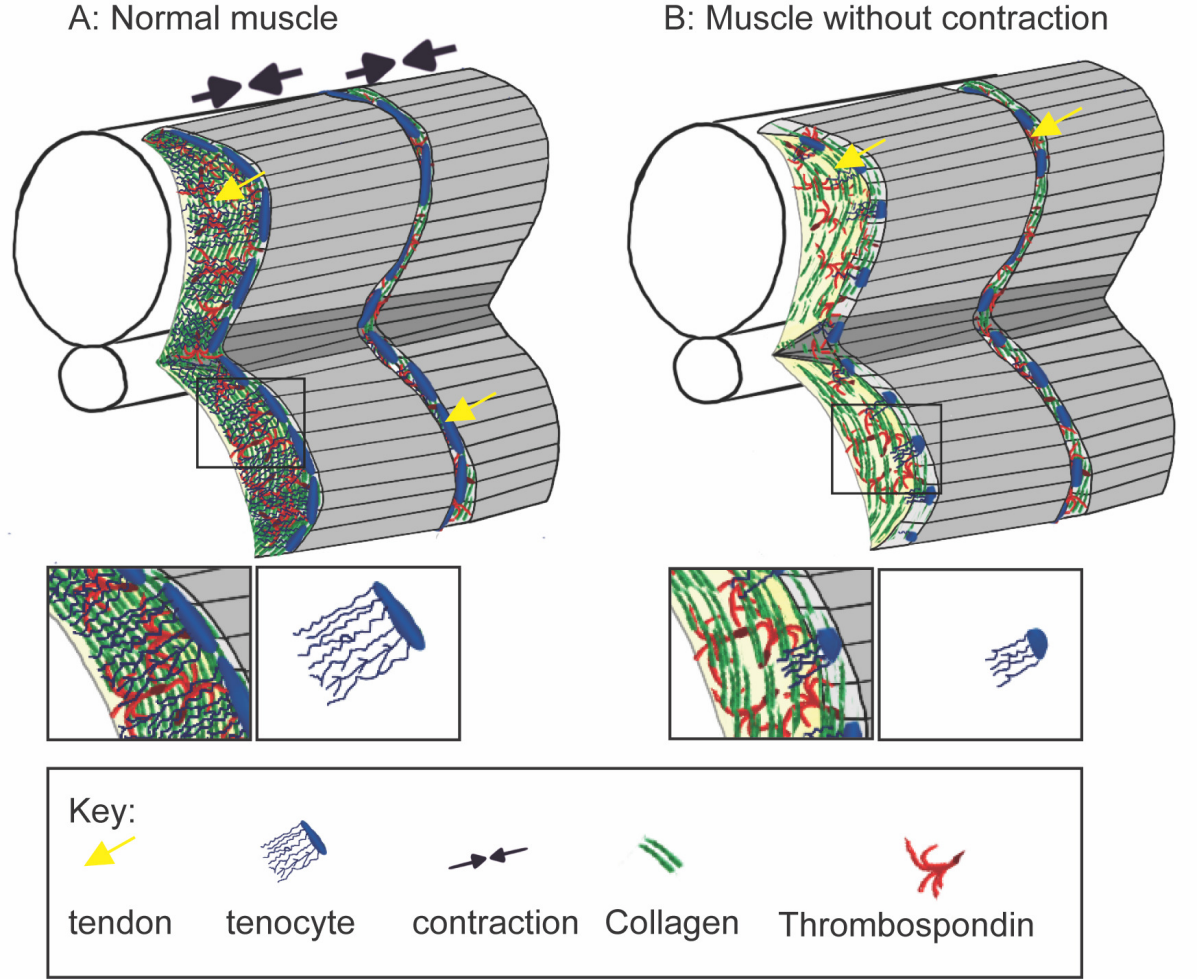
Muscle contraction is required for normal tendon development. (**A**) During normal muscle development, contractions increase the amount of extracellular matrix proteins, including collagen (green) and Thrombospondin (red), deposited in the developing tendon. These muscle contractions are also required for normal BMP signaling that prompts tenocytes (tendon precursor cells, blue) to elongate and extend complex protrusions (inset panels). (**B**) In the absence of muscle contraction, extracellular matrix deposition is decreased and tenocytes are rounded with shorter and less complex protrusions.

Immobilized zebrafish embryos developed misaligned tenocytes with shorter and less complex protrusions. The concentration of the tendon ECM protein Thrombospondin, which plays a critical role in tendon development and muscle maintenance (Subramanian and Schilling, 2014), was also disrupted. However, using electrical stimulation to induce muscle contraction in the immobilized zebrafish restored normal tenocyte development and tendon ECM. These creative experiments clearly demonstrate that contractile muscle tissue is required for tendon development.

How do cells integrate mechanical force with biochemical signals? This is a fundamental question throughout cell biology, and Subramanian et al. now have a hypothesis for how this works in tenocytes. They found that contractile force produced by muscle promotes the activity of a signaling pathway called TGFbeta in the tenocytes. Furthermore, TGFbeta is required to form the tenocyte protrusions and to express tenocyte-specific genes, including the gene that produces Thrombospondin. This suggests that one role of the tenocyte protrusions is to secrete and organize the tendon ECM.

The results reported by Subramanian et al. represent a dramatic step forward in our understanding of how tendons respond to contractile force during development. It remains to be seen whether similar mechanisms underlie muscle–tendon dynamics during tendon repair. Could electrical stimulation help tendons to repair themselves? Another major cause of tendon injury is the tendon ECM becoming less elastic with age, leaving one to wonder whether increasing Thrombospondin levels or activating TGFbeta signaling could rescue elasticity. If so, it is worth noting that the UC Irvine team had previously shown that the defects seen in Thrombospondin-deficient zebrafish can be rescued by expressing the human form of the protein (Subramanian and Schilling, 2014). This result indicates that zebrafish and human Thrombospondin work interchangeably, which will allow researchers to use the zebrafish model to investigate the therapeutic potential of this protein.
